# Alzheimer’s Disease Early Detection Using a Low Cost Three-Dimensional Densenet-121 Architecture

**DOI:** 10.1007/978-3-030-51517-1_1

**Published:** 2020-05-31

**Authors:** Braulio Solano-Rojas, Ricardo Villalón-Fonseca, Gabriela Marín-Raventós

**Affiliations:** 8 Digital Research Centre of Sfax, Sfax, Tunisia; 9 Institut Mines-Télécom, CNRS, Paris, France; 10 Université de Sherbrooke, Sherbrooke, QC Canada; 11 Digital Research Centre of Sfax, Sfax, Tunisia; 12 University of Sfax, Sfax, Tunisia; CITIC - ECCI, Universidad de Costa Rica, San José, Costa Rica

**Keywords:** Alzheimer, Deep learning, MRI, Computer-aided detection, Computer-aided diagnosis

## Abstract

The objective of this work is to detect Alzheimer’s disease using Magnetic Resonance Imaging. For this, we use a three-dimensional densenet-121 architecture. With the use of only freely available tools, we obtain good results: a deep neural network showing metrics of 87% accuracy, 87% sensitivity (micro-average), 88% specificity (micro-average), and 92% AUROC (micro-average) for the task of classifying five different classes (disease stages). The use of tools available for free means that this work can be replicated in developing countries.

## Introduction

Alzheimer’s Disease (AD) is the most common form of dementia among older adults [17]. It is a neurodegenerative disease without a cure. Its early detection is crucial because it allows those people who are going to be affected to prepare for future changes [17]. For example, some medications delay the disease. Also, their relatives can prepare and train for the care that will be necessary [17].

Early detection is not easy. One of the difficulties is the performance of people working at the clinic. People making a diagnosis are affected by several factors such as fatigue, stress, distractions, and inherent cognitive biases to specific conditions of the disease. When radiologists see a medical image, such as a magnetic resonance imaging (MRI), biased reasoning about the conditions of the disease will result in the loss of the opportunity to detect it. Graber et al. [7] found that about 74% of diagnostic errors are attributed to cognitive factors. Lee et al. [14] state that approximately 75% of all medical errors made were due to diagnostic errors by radiologists. A high workload, stress, fatigue, cognitive bias, and an inadequate system are part of the causal factors. Medical errors contrast with the fact that recently artificial intelligence, in particular, deep artificial neural networks (DNNs) have shown superhuman abilities in the detection of diseases in medical computer vision, as in the work of Rajpurkar [18]. We can design DNNs to integrate them into computer-aided diagnosis protocols for the detection of many priority diseases. One of these possible diseases is AD.

Currently, there is a body of images of healthy patients and patients with AD that is available through the database Alzheimer Disease Neuroimaging Initiative (ADNI)^1^. ADNI launched in 2003 as a public and private initiative. The leadership belongs to the researcher Michael W. Weiner. The main objective of ADNI has been to test whether medical images, other biomarkers, and clinical and neuropsychological evaluation can be combined to measure the progress of AD. The early detection of AD employing software would allow us to strengthen and improve medical protocols by providing what we call Computer-Aided Diagnosis (CAD).

As we commented, DNNs have become increasingly important and useful in recent years. One kind of these type of neural network is Convolutional Neural Networks (CNN). CNNs are inspired by the biological visual cortex and are used in areas as diverse as smart surveillance and monitoring, health and medicine, sports and recreation, robotics, drones, and self-driving cars [12].

This work consists of measuring the accuracy of the detection of Alzheimer’s disease of a three-dimensional CNN architecture, specifically a densenet-121, trained using the ADNI MRI images. We also have a low-cost economic objective. We aim to provide a technological artifact that has the potential of being used in the public health and wellbeing of citizens all over the world, in particular, for developing countries that have difficulties in accessing specialized hardware platforms for computation.

Before presenting the results of developing a low-cost densenet for Alzheimer’s disease detection, we first provide in Sect. 2 some background definitions to support our work. In Sect. 3 we describe previous work with more detail. Then in the next section, we provide the methodology used to realize this work. We present in Sect. 5 the results of the design chose. Finally, we analyze those results with concluding remarks and future work in Sect. 6.

## Background

We start with a short review of medical vocabulary used to provide a context for our research. First, we introduce different clinical stages of disease that we want to classify, and later, we describe two types of medical imaging used in the detection and diagnosis of AD.

### Clinical Disease Stages

There are different stages before the clinical diagnosis of AD. These are cognitively normal, significant memory concern, and mild cognitive impairment.

**Cognitive Normal (CN).** CN patients are the control subjects in the ADNI study. They have healthy aging. They show no signs of depression, mild cognitive impairment, or dementia [1].

**Significant Memory Concern (SMC).** SMC is a self-report significant memory concern from the patient, quantified by using the Cognitive Change Index and the Clinical Dementia Rating (CDR) of zero. Subjective memory concerns are correlated with a higher likelihood of progression, thereby minimizing the stratification of risk among normal controls and addressing the gap between healthy elderly controls and mild cognitive impairment. However, SMC patients score within the normal range for cognition [1].

**Mild Cognitive Impairment (MCI).** MCI participants have reported a subjective memory concern either autonomously or via an informant or clinician. However, daily living activities are mainly preserved, there are no significant levels of impairment in other cognitive domains, and no signs of dementia exist. Levels of MCI (early or late) are determined using the Wechsler Memory Scale Logical Memory II [1].

**Alzheimer’s Disease.** AD is the most common cause of dementia, a general term for memory loss and other cognitive abilities severe enough to interfere with daily life. It is a progressive disease, where dementia symptoms gradually worsen over several years. Individuals lose the ability to carry on a conversation and respond to their environment. Current medications cannot stop the disease from progressing, they can temporarily slow the worsening of dementia symptoms and improve quality of life for those with AD and their caregivers [17].

Since we aim to assess if those stages, including AD, are detected on medical imaging, particularly on Magnetic Resonance Imaging, we continue describing two medical imaging techniques.

### Medical Imaging

Medical imaging is the process and technique of creating visual representations of the inner of a human body for clinical analysis and medical intervention. We introduce two types of medical imaging. We are especially interested in the input of Magnetic Resonance Imaging (MRI) on DNN. Moreover, we also mention Positron Emission Tomography (PET) because it is sometimes an input that accompanies MRI. We follow describing what MRI and PET are.

**Magnetic Resonance Imaging.** MRI is a non-invasive imaging technology that produces three-dimensional detailed anatomical images without the use of radiation that damages human tissues. It is often used for disease detection and diagnosis and treatment monitoring. It is based on sophisticated technology that excites and detects the change in the direction of the rotational axis of protons found in the water that makes up living tissues [15].

**Positron Emission Tomography.** PET scans use radiopharmaceuticals to create three-dimensional images. These types of scans produce small particles called positrons. A positron is a particle with roughly the same mass as an electron but oppositely charged. Positrons react with electrons in the body, and when these two particles combine, they annihilate each other. This annihilation produces a small amount of energy in the form of two photons that shoot off in opposite directions. The detectors in the PET scanner measure these photons and use this information to create images of internal organs [16].

## Previous Work

Our literature review assesses how much progress has been made and what can be contributed in the detection of AD using deep learning, in particular with Convolutional Neural Networks (CNN). We only focus on AD however detection of another neurodegenerative disease using DNNs has been investigated [13, 19].

We used IEEE^2^ as the source for Artificial Neural Networks because, according to Journal Rankings^3^ on the category of Artificial Intelligence, IEEE is the first on both SJR and H-Index sortings. We used the search engines Duck Duck Go, and Google Scholar to find illustrative publications.

We used the search string “deep AND learning AND alzheimer AND mri” in order to assess the use of convolutional deep learning in our application of interest. We ran the query mentioned from 2016 to the present (in 2019) since we are searching about recent advancements in neural networks. We retrieved from IEEE Digital Library 81 records with this query, including conferences, journals, and early access articles.

We screened by title, and if the title was too ambiguous by abstract. We searched for the application of convolutional deep learning and we obtained 32 articles. Notably, we searched for literature that included the design of convolutional deep learning artifacts for computer vision to detect AD in MRI and other modalities. Besides, the literature was restricted to supervised learning. For example, we did not include convolutional autoencoders alone.

For the articles we deemed appropriate, we developed a data extraction spreadsheet to serve for analysis where we collected the following information about each publication: (1) year of the paper, (2) architecture of the neural network, (3) if the MRI images were processed, (5) the modalities (number of inputs), (6) the number of classes used, and the metrics of (7) accuracy, (8) sensitivity, (9) specificity, and finally (9) the Area Under the curve Receiver Operating Characteristics (AUROC).

In this literature review, with our data extraction spreadsheet, we find a severe problem. Almost 50% of papers report accuracy but do not report sensitivity, specificity or AUROC. Accuracy alone can be misleading. A classifier can report a high accuracy and yet have a low capacity of true prediction. We also conclude that the studies are too diverse to allow a meaningful comparison. It seems that there is a race to obtain greater accuracy, although this metric is misleading. In addition, multiclass classification is avoided. Most studies implement one-vs-one classifiers, thus achieving higher accuracy values. When the number of classes increases the accuracy tends to decrease. In fact, we did not find any article with multiclass classification with more than four classes. Nor did we find many articles that used the densenet architecture. Only three papers used densenets, of which two [6, 9] are three-dimensional but with shallow densenets and one [11] uses deep densenets but two-dimensional. Finally, the quantitative analysis of the collected items does not generate a great contribution due to these defects. However, in the review of the articles, we find articles of remarkable quality as [2]. We also consider that some of the papers collected are not repeatable.

In contrast to existing studies, we seek to create a multiclass neural network using only tools available for free. Besides, we do not give greater importance to accuracy over other metrics and analysis. Finally, we want our process to be repeatable, and we report it complete along with all the parameters used, as explained in the next sections.

## Methodology

In this section, we describe how we collect data using the ADNI study and how we preprocess these data. Then, we present the development carried out and how we produced, using the Google Collaboratory tool, an Alzheimer’s prediction model to fulfill the objective of measuring the accuracy of the detection of Alzheimer’s disease using a three-dimensional Densenet-121.

### Data Acquisition

In this work, we used the data from ADNI. We used their beta advanced search functionality with the following criteria. In Projects, we checked ADNI. In Research Group, we checked MCI, EMCI, AD, SMC, and CN. In Modality, we checked MRI. We only chose MRI and did not add PET because of economic restrictions. PET requires radiopharmaceuticals, as mentioned. It is more usual to find MRI in contexts of economic limitations. Continuing with search options, in Image Description, we used MPRAGE. In Acquisition Plane, we used SAGITTAL, and finally, in Weighting, we used T1. The rest of the search fields were left with their default values. With those parameters, we obtained 5556 magnetic resonance images with the following distribution: 1520 Cognitive Normal (CN), 186 Significant Memory Concern (SMC), 1222 Early Mild Cognitive Impairment (EMCI), 1274 Mild Cognitive Impairment (MCI), 636 Late Mild Cognitive Impairment, and 718 Alzheimer’s Disease.

The images obtained from ADNI are in Digital Imaging and Communication On Medicine (DICOM) format. The files are in a zipped archive of 55.5 GB, and the uncompressed files measure 138 GB. We reduce that size with data preprocessing, and we explain how and why in the next section.

### Data Preprocessing

MRI image data are groups of images. Every image is a slice, and the group of slices shapes the MRI. Every image or slice is a matrix of pixels. Each slice has an associated spatial thickness because they represent reality. Also, every pixel in every slice has a spacing, that is the space they represent. Thus, the data is volumetric or, in other words, rectangular cuboids. Taking that into consideration, we do the following transformations to the data. First, we convert all volumetric pixels (voxels) to a spacing of $1\times 1\times 1$ mm. This conversion may add or delete slices, or slice pixels. After that, we convert every slice to $256\times 256$ pixels as follows. Some slices are not square. If they are not, we fill in with black pixels. After they are square, if they are not $256\times 256$, we convert them to that size using interpolation. Concerning the size, we also make the cuboids have 256 slices using interpolation. The result is $256\times 256\times 256$ cubes. From these cubes, to keep “see” only the brain as would a human do, we make a cut from slice 40 to slice 214, from row 50 to row 199, and from column 40 to column 239. With that cut, we discard borders full of black pixels and conserve the inner cuboids that have useful information (the brain). Since we made all the MRI the same size, we assume that the cut keeps the brain and we do not have to apply techniques like image segmentation (cutting the brain using pattern recognition). From those cut cuboids, we use only half of the slices and half of the rows and columns of every slice by eliminating one in between for all. The latter reduces the size of the images and the dimensionality of the problem considerably. Last, we normalize the images pixel values to an interval of $-1.0$ to 1.0.

Data preprocessing can be done both online or beforehand. We implemented both. However, to maintain a low-cost objective, we use a script to apply the preprocessing previously to the task of neural network training, and we load the MRI data already transformed. The previous transformation may be done on a desktop or laptop computer. Although it will take hours, it is not a task that will take more than a day on current commodity hardware.

After data preprocessing the images occupy only 13.5 GB, we have reduced the size of the images slightly more than ten times. This reduction is beneficial to minimize neural network training time and storage needs of our development explained in the next section.

### Our Development

We chose to use a convolutional DNN of densenet-BC architecture because of our objective to use the least resources possible. This kind of architecture has an excellent performance with fewer parameters to train [10]. We based our development on the implementation of Hara et al. [8]. We used their densenet implementation for the densenet-121 architecture. This implementation, in turn, is based on the two-dimensional implementation available in the Pytorch code. The implementation of Hara et al., however, is not generic. It was made for video and incorporates the variables *sample_size* and *sample_duration* that have to do with the size and duration of video samples. We eliminated that and made the implementation general. It works with all kinds of cuboids. Also, we added a channels parameter because the implementation always considered 3 channels (usually red, green and blue colors), but the magnetic resonance images are monochromatic.

Using this implementation we configure the training process of the neural network with the following parameters.

**Table:** 

Training	We use 75% of the data obtained from ADNI as the training dataset. The data is obtained randomly from the complete data set
Batch size	For the phase of training, we use a batch size of 5 MRI based on experimental results by [2]
Testing	The testing dataset is the remaining 25% of the data
Channels	We send a parameter of 1 to the constructor of the neural network because the images are monochromatic
Classes	Initially, we sent a parameter of 6 to the constructor of the neural network. However, we decided to eliminate the SMC class because it is a subjective class. We consider it training noise. Finally, we use a parameter of 5 classes to classify
Dropout	We use a dropout rate of 0.7 based on observations by [2]. This prevents overfitting
Loss	We use a cross-entropy loss function. It is useful in classification problems that are not binary and, in our case, we have 5 or 6 classes
Optimizer	We use stochastic gradient descent (SGD). This popular optimizer is useful in the case of unbalanced data, which is our case
Learning	In the SGD optimizer, we use a learning rate parameter of 0.1 and a drop in the learning rate in the sixty epoch of 0.1. The latter reduces the learning rate to 0.01 in that epoch
Momentum	Since the SGD optimizer with momentum usually finds flatter local minima, we use a typical momentum of 0.9
Epochs	Since we use the Google Collaboratory platform, we set the maximum number of epochs to 80. It was not possible to exceed above 90 epochs to reach 100 epochs because the platform disconnects us before achieving it

With that parameters, we pushed the limits of the Google Colaboratory platform to produce a state-of-the-art DNN. Although other authors claim that the free-of-charge resources of Google Colaboratory “are far from enough to solve demanding real-world problems and are not scalable” [3], we use it as the platform that provides us Graphics Processing Unit (GPU) computation. This decision has limitations and implications. As explained in [3], there only 12 h of free use of the GPU backend. We have even noticed less sometimes, approximately 10 h. After that time, Google Colaboratory disconnects and deletes the virtual machine provided. If the user reconnects, the new machine supplied only offers 3 h of GPU backend. After that, it is not possible to connect to a backend with GPU for a determined number of hours. These limitations imply that the training and testing have to be done in one run before the first 12 h end. There are other implications to the restrictions. For instance, it is customary to test or validate neural networks during training; thus the loss and accuracy of the neural networks can be analyzed at each epoch. However, to reduce computation time, testing or validation of the trained DNN is only done at the end. We chose this because a validation cycle of 25% of the data takes approximately 2 or 3 min. In 30 epochs, that would take 1 h or more. This trade-off is not severe, we can save intermediate neural networks states and study them after finishing the training. However, this choice also implies that techniques like early stopping can not be employed. There are also disk size limitations.

Taking all the limitations into account and with the mentioned configuration parameters of our development, we obtained the results that we discuss in the next section.

## Results and Discussion

**Fig. 1.**
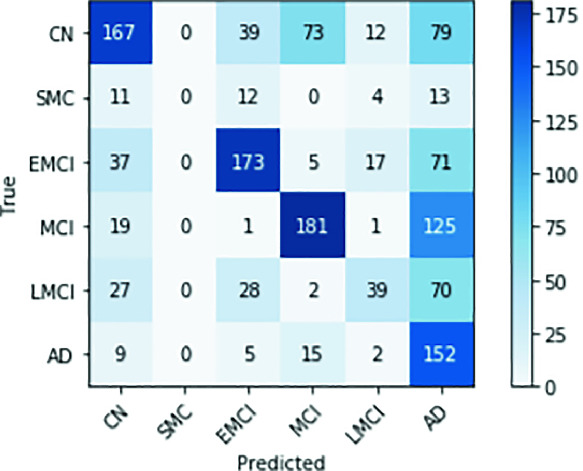
Confusion matrix with the SMC class at 50 epoch

The first finding of this work is the characterization of the significant memory concern class as a noisy class for training. This problem may be due to the fact that the class is subjective and is possibly composed of at least two classes: those who will develop the disease and those who will not. Also, those who will develop it may have different levels of progression, being, in turn, a class composed of different classes. Another reason for the class to be problematic is its size. It is the smallest cohort and by far. This makes it difficult to classify during training. In the Fig. 1, we show how this class is not classified after 50 training epochs. As seen, the column of the predicted SMC class is full of zeros. It is also notable that the other classes already have a good level of correct classification. We decided to remove this class from the data set. This reduced the total data set from 5556 MRI to 5370 MRI.

**Fig. 2.**
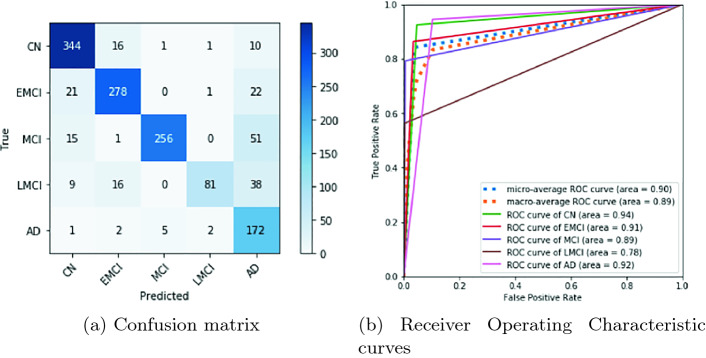
Metrics of evaluation of the densenet-121 at 80 epochs

After eliminating the SMC class and training for 80 epochs, we obtain a neural network with good classification metrics of the five remaining classes. The results can be seen in Figs. 2a and 2b. In Fig. 2a, the confusion matrix, we can see how most values are kept diagonally. There are a certain amount of incorrect predictions. However, there is an interesting, unexpected feature. These incorrect predictions are mostly pessimistic; that is, there are more errors above the diagonal that under it, and this means that the classifier is making errors that put the prediction on upper disease stages. This is clearly in favor of patients because, in terms of diagnosis of diseases, a false positive is better than a false negative. Figure 2b shows the quality of our classifier for each class and all classes together. As the area under each curve approaches the value 1.0, greater diagnostic ability of the classifier is demonstrated. It is clear that, although our classifier is not perfect, it is a good one.

**Fig. 3.**
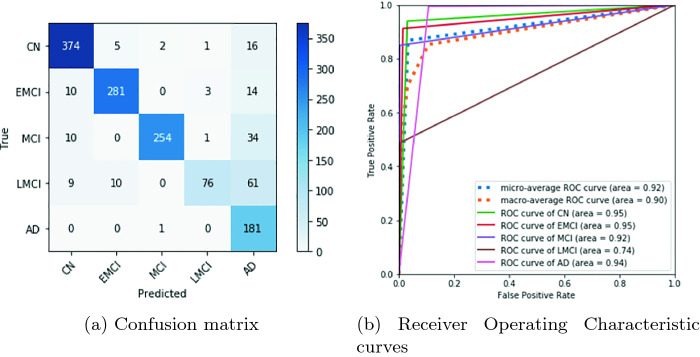
Metrics of evaluation of the densenet-121 at 110 epochs

Although we obtained an already good predictive model, we wanted to improve it using the same tools we already used. However, because we use Google Colaboratory, we could not repeat the process of training and add a significative number of epochs. Therefore, we saved the model at 80 epochs. Then, after waiting 12 h because of the Google Colaboratory restrictions, we restarted the process of training again from the 80th epoch and pushed it to 110 final epochs. The predictive performance of this new model can be seen in Figs. 3a and 3b.

This new confusion matrix (Fig. 3a) and ROC curve plot (Fig. 3b) show that it is possible to improve the prediction model even under the restrictions of free-of-charge resources like Google Colaboratory. We may notice that as we improve all classes, the Late Mild Cognitive Impairment class gets worse in the prediction. That is, we approach a local minima solution that improves the classes in general but moves away from the correct prediction of the LMCI class. We believe that this effect is due to the lack of balance in the data. LMCI is the class with the least amount of data after we removed Significant Memory Concern. This can be solved with data augmentation as done, for instance, in [4]. However, if we do this, we would reduce the amount of maximum epochs that we can use during training. However, although LMCI does not have the best classification, it is classified pessimistically, then we can accept the commitment of not balancing the data. We include more prediction performance metrics of this last model in Table 1.

**Table 1.**
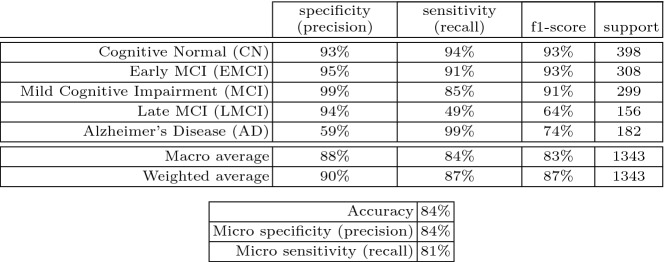
Metrics of the obtained DNN at 110 epochs

As we can see in Table 1, the worst figures are the specificity of Alzheimer’s Disease and the sensitivity of Late Mild Cognitive Impairment. We could also include the sensitivity of Mild Cognitive Impairment in the bad numbers, although the percentage of recall is not poor. The poor specificity of Alzheimer’s is acceptable because it reaches almost 100% sensitivity or recall. The number is bad because other classes are classified as AD, but in a context of pattern recognition that always has risks and costs, it is in favor because it is pessimistic and in medicine that can reduce risk and future costs. In the same manner, the bad number of LMCI is also acceptable because the class is mostly classified as AD. Therefore, considering the economic restrictions, the final figures of 84% accuracy, 84% specificity (micro) and 81% sensitivity (micro) are acceptable. We chose to report final micro-average figures instead of macro-average because in a multi-class classification setup, micro-average is preferable when there is a class imbalance. However, as it can be noticed the macro average and the weighted average are better.

## Conclusions and Future Work

The use of free-of-charge resources limited this study. With this restriction, we explored a low-cost way to generate a deep artificial neural network that shows good performance metrics. We demonstrate that the model can still be improved. This prediction model can be useful in developing countries if user interface and interpretation are added and it has the potential of being used in remote medicine contexts.

In the future, we want to create a user interface for the diagnosis of AD. We can do this based on the implementation of Chester [5], a computerized chest X-ray disease prediction system that is delivered on the web. With the recent creation of tools such as ONNX and TensorFlow.js, PyTorch-trained models can be converted to work in the browser and compute using WebGL [5]. This interface would have not only prediction but also interpretation or explanation through relevance maps or heat maps.

Last, to contribute to reproducibility and transparency in academic work, we provide the source code of our DNN at https://github.com/bsolano/Alzheimer-ResNets.

## Footnotes

1 http://adni.loni.usc.edu.

2 https://ieeexplore.ieee.org/.

3 https://www.scimagojr.com/.
